# Performance of Different Saccharomyces Strains on Secondary Fermentation during the Production of Beer

**DOI:** 10.3390/foods13162593

**Published:** 2024-08-19

**Authors:** Brooke A. Dilmetz, Gurpreet Brar, Christopher T. Desire, Jon Meneses, Manuela Klingler-Hoffmann, Clifford Young, Peter Hoffmann

**Affiliations:** 1 Clinical & Health Sciences, University of South Australia, Adelaide, SA 5000, Australia; 2 Coopers Brewery Ltd., Regency Park, SA 5010, Australiajonm@coopers.com.au (J.M.); 3 Future Industries Institute, University of South Australia, Mawson Lakes, SA 5095, Australia

**Keywords:** *Saccharomyces cerevisiae*, bottle conditioning, beer, proteomics, viability, secondary fermentation

## Abstract

Bottle conditioning of beer is an additional fermentation step where yeast and fermentable extract are added to the beer for carbonation. During this process, yeast must overcome environmental stresses to ensure sufficient fermentation in the bottle. Additionally, the yeast must be able to survive for a prolonged time, as a decline in viability will lead to alterations in the product. Here, we investigated the effects of bottle conditioning on beer using six different yeast strains from the brewing, wine making, and distilling industries over 120 days. The ale and lager strains resulted in a beer possessing typical characteristics of a pale ale-style beer, whereas sparkling wine and distilling yeast strains resulted in aromas that were uncharacteristic, which was expected. In addition, we observed that the various strains had different propensities to survive during bottle conditioning. Proteomic analysis was performed to ascertain protein abundance changes and reveal biological processes that potentially enabled specific yeast strains to survive longer during secondary fermentation. Our results showed that proteins associated with oxidoreductase activity and mitochondrial ribosomes were increased in the yeast strain with superior survival and were able to respond to cellular stress more effectively, whereas proteins associated with cell wall modulation were increased in the strain with poor survival characteristics. Overall, we demonstrated the impact of yeast selection on bottle conditioning and the biological processes involved in yeast physiology under these conditions.

## 1. Introduction

Bottle conditioning, which is also known as beer refermentation, is a commonly used process among breweries in Belgium, the UK, and the USA [1]. This process involves the inoculation of yeast into mature beer that has been primed with a fermentable extract such as sugar, where the yeast performs a secondary fermentation for one to two weeks [2]. During bottle conditioning the yeast naturally carbonates the beer and improves flavour stability [3]. Flavour stability is mainly improved as the yeast reduces the formation of ageing compounds such as (E)-2-nonenal and diacetyl [4,5].

Yeast has four distinct growth stages during secondary fermentation: a lag phase, a growth phase, a stationary phase, and a death phase [3]. Ideally, the lag phase should be non-existent or minimal during bottle conditioning as it is at this time that the risk of microbial contamination is highest [3]. The yeast subsequently enters the growth phase where they rapidly divide, consume the available fermentable extract, and produce ethanol, carbon dioxide, and other aromatic compounds [6]. Yeast growth slows as the concentration of fermentable extract declines and the yeast enter the stationary phase. After a prolonged period in the stationary phase, the viability of the yeast declines.

The positive influence of yeast on flavour stability during bottle conditioning relies on the yeast maintaining viability. As viability declines, the yeast undergoes autolysis, which results in the accumulation of intracellular compounds such as fatty acids and proteases in the beer, ultimately impacting the flavour and foam stability [7,8]. Beer is a sub-optimal environment for yeast growth as it is nutrient poor, has low pH, a high ethanol concentration, carbon dioxide pressure, and experiences changes in temperature during transport and storage [6]. It is likely that the ability of the yeast to withstand these conditions is imperative to maintaining beer quality and improving shelf-life characteristics. Therefore, it is crucial to select a yeast strain that has an increased lifespan during these conditions and does not compromise the sensory aroma of the beer.

Bottle conditioning is also used to produce sparkling wines [9]. The capacity of the yeasts to survive in this environment have also been considered important to ensure successful bottle conditioning in sparkling wine production [10,11]. Additionally, a transcriptomic study reported that despite some similarities to primary fermentation, metabolic processes in yeast such as low temperature and carbon dioxide pressure were specific to secondary fermentation [9].

The aim of this study was to compare the viability and impact of different yeast strains on bottle-conditioned beer and to investigate the biological processes that underpin their ability to survive. Here, we analysed the impact of yeast strains utilised in the brewing, wine, and distilling industries, and their influence on different attributes of the beer, such as alcohol concentration, apparent extract, foam stability, and aroma during bottle conditioning over four months. Volatile and sensory analysis for the different yeast strains is important as changes to the aroma can impact the product and consumer preference. The viability of the yeast during prolonged bottle conditioning was also evaluated. Proteomic analysis was performed to gain a better understanding of the underlying biological process that enabled certain yeast strains to survive longer during bottle conditioning. Biological processes associated with oxidoreductase metabolism were upregulated in the strain with good survival characteristics, whereas processes associated with cell wall organisation were downregulated in yeast that had poor survival during bottle conditioning.

## 2. Materials and Methods

### 2.1. Yeast Strains, Media, and Culture Conditions

The six Saccharomyces strains were obtained from different fermentation industries such as brewing, wine making, and distilling (Table 1).

Ale brewing yeast 1 and Lager brewing yeast 1 were streaked onto Wallerstein laboratory nutrient agar (WLN-A) (Part number: PP2137, Thermo Fisher Scientific, Waltham, MA, USA) and incubated at 25 °C for 5 days. Ale brewing yeast 2 was isolated by filtration of a commercial beer with a 0.2 µm filter and incubated at 25 °C for 5 days on WLN-A. Colonies were re-streaked onto WLN-A and incubated at 25 °C for 5 days. Approximately 1 g of the dried yeasts (Lager brewing yeast 2, Sparkling wine yeast, and Distilling yeast) were added to 10 mL of sterile water and incubated at 30 °C for 30 min. The yeast was diluted, spread onto WLN-A, and incubated at 25 °C for 5 days. All yeast strains were maintained at 4 °C.

Wort was prepared from a concentrate (Coopers Brewery Ltd., Regency Park, Australia) and made up to a gravity of 12.35 ± 0.04 °P before autoclaving. Two biological trials for each yeast strain were carried out by inoculating colonies of each yeast strain into 15 mL of wort in disposable 50 mL sterile screw cap containers. The yeast cultures were incubated at 20 °C with shaking at 170 rpm for 24 h. The cultures were transferred to 150 mL of wort in 250 mL glass bottles and incubated at 20 °C for 24 h. Afterwards, the 150 mL cultures were transferred to 1500 mL of wort in 2000 mL glass shake flasks that were plugged with cotton wool and incubated at 20 °C for 72 h.

Pale ale beer was primed with 0.5 °P sucrose and then capped in the brewery. The beer was uncapped, inoculated with yeast at approximately 0.2 × 10^6^–1.0 × 10^6^ cells/mL, and then recapped. Beer was stored at 20–22 °C for 1 day, 14 days, 30 days, 60 days, 90 days, and 120 days before opening.

### 2.2. Cell Concentration and Viability Determination

Cell concentration and viability was determined using a Cellometer X2 Fluorescent Viability Counter (Nexcelom Bioscience LLC, Lawrence, MA, USA) and the ViaStain™ Yeast Kit for Live/Dead Concentration (PerkinElmer, Mulgrave, Victoria) as previously described [12]. Briefly, beer samples were tossed 20× to remove gas and then 50 mL of beer was centrifuged at 3000× *g* for 5 min. The supernatant was removed, the cell pellet resuspended in yeast dilution buffer and then an equal volume of ViaStain™ was added. Samples were counted in triplicate.

### 2.3. Vitality Determination

The vitality of the different yeast strains during propagation in wort was performed using resazurin as previously described [13].

### 2.4. Foam Stability

Foam stability was determined using a Haffmans NIBEM-TPH Foam Stability Tester (Pentair, London, UK) as previously described [12].

### 2.5. Apparent Extract (AE), Alcohol Concentration, and pH Measurement

AE, alcohol concentration, and pH measurement were performed as previously described [12].

### 2.6. Volatile Analysis

Standard solutions were prepared by accurately weighing amounts (± 0.001 g) of chemical standard (Merck, Darmstadt, Germany) and dissolving compounds in ethanol (Wilmar, Yaraville, Victoria). Standard solution A was prepared by the addition of 2-methyl-1-butanol (2.50 g), 3-methyl-1-butanol (2.50 g), isobutanol (1.25 g), propanol (1.25 g), and acetaldehyde (2.5 g) in 50 mL of ethanol. Standard solution B was prepared by the addition of ethyl hexanoate (0.5 g) and dimethyl sulphide (DMS) (0.1 g) in 50 mL of ethanol. Standard solution C was prepared by the addition of isoamyl acetate (0.25 g) and ethyl acetate (5.00 g) in 50 mL of ethanol. Standard solution D was prepared by the addition of 10 mL of standard solution A, 10 mL of standard solution B, and 5 mL of standard solution C and made up to 200 mL with ethanol. Calibration standards were prepared by diluting 1–10 mL of standard solution D in 0–9 mL of ethanol. Four mL of each calibration standard was made up to one hundred mL with RO water. The internal standard was a solution of ethyl methyl sulphide (100 µg/L) for DMS and 3-heptanone (1.6 mg/mL) in ethanol. Both samples and standard solutions were stored refrigerated at 4 °C.

Beer samples were stored at 4 °C for at least 12 h. Autosampler vials containing sodium chloride (2 ± 0.1 g) (Chem-Supply, Gillman, SA, Australia) were cooled to 0–4 °C. Five mL of cooled beer was added to the autosampler vial, followed by 50 µL of each of the two internal standards. Autosampler vials were capped immediately, agitated for 30 s, and then equilibrated in the autosampler for at least 30 min prior to injection.

An Agilent 6890 N gas chromatograph (GC) (Agilent Technologies, Waldbronn, Germany) with a Zebron ZB-Wax_Plus_ column (30 m, 320 µm i.d., 0.5 µm film thickness) (Phenomenex, Torrance, CA, USA) was connected to an Agilent G1888 Headspace sampler (Agilent Technologies) and a Flame Ionisation Detector (FID). The FID temperature was 200 °C with a hydrogen and air flow of 30 mL/min and 300 mL/min, respectively. An inlet split mode (ratio 30:1) and injection temperature of 200 °C was utilised. The flow rate of the carrier gas (nitrogen) was 30 mL/min. The temperature program was as follows: 43 °C for 4 min, raised to 160 °C at 10 °C/min for 12 min, and then reduced to 43 °C at 40 °C/min for 3 min. The ChemStation OpenLabs CDS (Rev C.01.07 (27)) software package for GC control and data integration (Agilent Technologies) was used.

### 2.7. Sensory Evaluation of Beer

The aroma of bottle-conditioned beers (14 days, 30 days, 90 days and 120 days) was evaluated by a trained panel comprised of a minimum of five tasters. All tasters were employees of Coopers Brewery Ltd. who possess extensive experience in sensory evaluation. Blind tasting was conducted on all experimental samples, with the trained panel asked to comment on the sensory aroma of the presented sample in comparison to a typical pale ale profile. Ethical review and approval were waived for this study due to the evaluation being performed using standard practices at a commercial brewery.

### 2.8. Sample Preparation for Proteomic Analysis

Yeast cells were collected from beer by centrifugation at 3000× *g* for 5 min. Pellets were washed with ultrapure water, centrifuged at 3000× *g* for 5 min, and then immediately frozen on dry ice. When required, cell pellets (1 × 10^7^ cells) were defrosted on ice. Cells were lysed in 4% *w/v* sodium dodecyl sulfate (Fisher BioReagents, Fair Lawn, NJ, USA)/50 mM Tris-HCl (pH 7.4) (Sigma-Aldrich, St. Louis, USA) buffer. Cells were added to 50 ± 5 mg of zirconium oxide beads (BioTools Pty Ltd, Brendale, Australia) and homogenised using a Next Advance Bullet Blender Storm 24 (BioTools Pty Ltd.) on level 10 for 3 min. Sample debris was removed by centrifugation at 20,000× *g* for 5 min at room temperature and the supernatant transferred to a fresh eppendorf. Proteins were precipitated using 4× ice-cold (−20 °C) acetone (Chem-Supply) overnight. Proteins were isolated by centrifugation at 20,000× *g* for 10 min at −9 °C, washed using ice-cold acetone and centrifuged at 20,000× *g* for 10 min at −9 °C, and then dried on ice for 5 min. Proteins were resuspended in 8 M urea (Sigma-Aldrich)/50 mM ammonium bicarbonate (Sigma-Aldrich) and then quantified using tryptophan fluorescence [14]. Proteins were reduced using dithiothreitol (final concentration 10 mM) (Sigma-Aldrich) and incubated at room temperature for 60 min with shaking at 300 rpm. Proteins were alkylated using 2-chloroacetamide (final concentration 15 mM) (Sigma-Aldrich) and incubated in the dark at room temperature for 30 min with shaking at 300 rpm. Samples were diluted with 50 mM ammonium bicarbonate to achieve a urea concentration of ≤0.8 M. Trypsin Gold (Promega Corporation, Madison, WI, USA) was added to achieve a 1:50 (enzyme/protein) ratio. Samples were incubated at 37 °C overnight with shaking at 500 rpm. Samples were acidified using 100% formic acid (Fisher Scientific, Pardubice, Czech Republic) to obtain a pH of approximately 2–3 and then desalted using Sep-Pak^®^ Vac 1cc (50 mg) C18 cartridges (Waters Corporation, Milford, MA, USA). Samples were subsequently dried to completion under vacuum at 50 °C. Peptides were resuspended in 0.1% formic acid and quantified using tryptophan fluorescence prior to liquid chromatography tandem mass spectrometry (LC-MS/MS).

### 2.9. LC-MS/MS

Samples were analysed using an ACQUITY UPLC M-Class system (Waters Corporation, Milford, USA) connected to a ZenoTOF 7600 mass spectrometer with an OptiFlow Turbo V ion source (SCIEX, Singapore, Singapore). Approximately 200 ng of peptides were loaded onto a nanoEase M/Z HSS T3 reversed phase C18 column (100 Å pore size, 1.8 µm particle size, 300 µm i.d. × 150 mm) (Waters Corporation) heated to 40 °C at a flow rate of 5 µL/min. Buffer A was 99.9% water and 0.1% formic acid and Buffer B was 99.9% acetonitrile (Merck, Burlington, MA, USA) and 0.1% formic acid. Peptides were separated with a gradient of 3–35% buffer B over 45 min. Other MS settings include ion source gas 1 and 2 set to 20 and 15 psi, respectively; curtain gas 35 psi; collision gas (CAD) 7; source temperature 150 °C; and the spray voltage set to 5000 V. A Zeno Sequential Window Acquisition of All Theoretical Mass Spectra data independent acquisition (Zeno SWATH-DIA) scheme (64 variable-size windows covering a 400–1500 m/z precursor mass range) was performed with an 0.1 s accumulation time, 80 V declustering potential, and 10 V collision energy. Raw data were acquired using SCIEX OS (v3.1.0).

### 2.10. Data Processing and Analysis

Proteomic data were analysed using Spectronaut 18 (Biognosys, Schlieren, Switzerland) [15] using the directDIA+™ workflow. Standard settings included enzyme, Trypsin/P; fixed modifications, carbamidomethyl (C); variable modifications, acetyl (Protein N-term) and oxidation (M); false discovery rate, 1%. Mass spectra were searched against the *Saccharomyces cerevisiae* FASTA database from UniProt (yeast: UP000002311_559292, 6060 entries, 15 March 2023). The mass spectrometry proteomics data have been deposited to the ProteomeXchange Consortium [16] via the PRIDE [17] partner repository with the dataset identifier PXD053930. GO terms were assessed using the tool “GO Term finder” from SGD database [18] and GO terms with an adj. *p*-value ≤ 0.01 were selected. Redundant terms were removed.

Data visualisation and statistical analysis for non-proteomic data was performed in GraphPad Prism 10 (v10.0.2) using an ordinary, one-way ANOVA.

## 3. Results and Discussion

In this study we evaluated six yeast strains that are used in the commercial brewing, sparkling wine, and distilling industries on their ability to perform secondary fermentation in beer, their long-term viability, and impact on the final product. The yeast strains were grown in wort until they reached post-diauxic/stationary phase, prior to seeding for bottle conditioning. The metabolic activity of the different yeasts during propagation was assessed using resazurin, where they had entered the post-diauxic or stationary phase after 72 h (Supplementary Figure S1). It has been previously shown that as the yeast enters the stationary phase, there is minimal resazurin production [13]. Yeast strains were grown to the post-diauxic or stationary phase to ensure they were seeded at a similar growth period and to potentially improve their stress tolerance [12]. We observed an increase in fluorescence at 24 h compared to 4 h and 72 h for Ale brewing yeast 2, which was due to a large outlier and not due to biological changes in the yeast. Compared to the brewing strains, the Sparkling wine and Distilling yeast strains did not readily ferment the wort and produced a higher apparent extract (AE), as well as a lower alcohol concentration after 72 h propagation (Supplementary Table S1). As maltose and maltotriose are the most abundant sugars in the wort, the Sparkling wine and Distilling yeasts may be less efficient in metabolising this sugar [19]. Additionally, the viability of the different yeast strains was between 88% and 99% at the time of seeding for bottle conditioning (Supplementary Figure S2). The different yeast strains had good viability for bottle conditioning despite their differences in fermentation ability.

### 3.1. Growth of Yeast Strains during Bottle Conditioning

**Figure 1:**
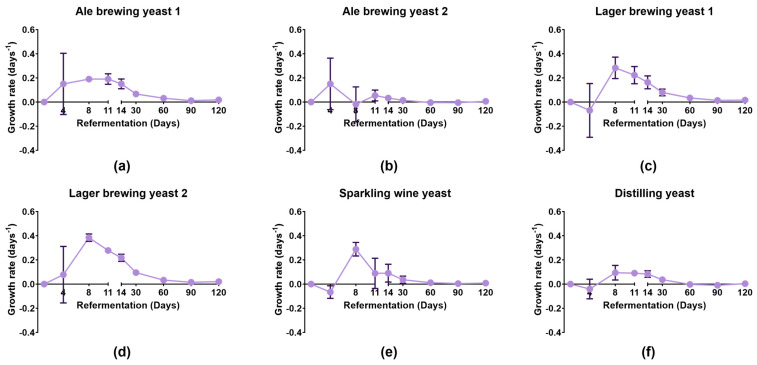
Growth rate of different yeast strains during bottle conditioning. Yeast strains were seeded into pale ale beer that was primed with 0.5 °P fermentable extract and incubated at 20–22 °C for 120 days. Growth rates were determined for (**a**) Ale brewing yeast 1, (**b**) Ale brewing yeast 2, (**c**) Lager brewing yeast 1, (**d**) Lager brewing yeast 2, (**e**) Sparkling wine yeast, and (**f**) Distilling yeast. Data shown as the mean growth rate and error bars represent standard deviation of biological replicates.

The growth rate of the different yeast strains during bottle conditioning of beer were compared using cell counts (Figure 1 and Supplementary Figure S3).

Lager brewing yeast 1, Lager brewing yeast 2, and Sparkling wine yeast had the fastest growth rate which reached a maximum of 0.28 ± 0.09 days^−1^, 0.38 ± 0.03 days^−1^, and 0.29 ± 0.06 days^−1^, respectively, on day 8. Lager brewing yeast 1, Lager brewing yeast 2, and Sparkling wine yeast strains had a decline in growth rate after day 8 of secondary fermentation. In contrast, all other strains had a decline in growth rate after 14 days. This indicated that Lager brewing yeast 1, Lager brewing yeast 2, and Sparkling wine yeast completed secondary fermentation faster than the other strains. The improved growth of the Sparkling wine and Distilling yeast strains during bottle conditioning compared to propagation was most likely due to the main fermentable extract being sucrose instead of maltose. It has been previously shown that glucose, fructose, and sucrose were easily fermented compared to maltose and maltotriose during bottle conditioning [20]. The slower decline in growth rate for the other yeast strains suggested that these strains may utilise other sugars to continue their growth compared to the Sparkling wine yeast. Ale brewing yeast 2 had a low growth rate throughout bottle conditioning. Despite this, it seemed that Ale brewing yeast 2 was able to successfully complete bottle conditioning within 30 days, as discussed in the following sections.

### 3.2. Impact of Yeast Strain on Beer Characteristics

**Figure 2:**
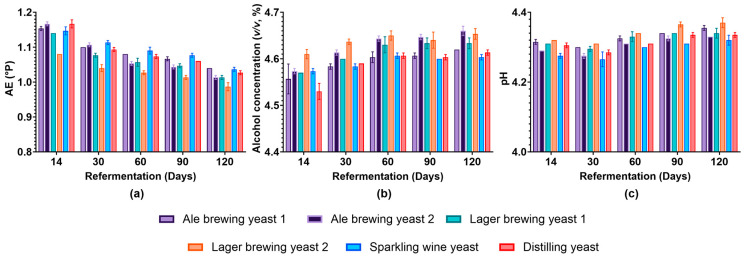
Evaluation of beer characteristics for the different yeast strains during bottle conditioning. (**a**) AE (°P), (**b**) alcohol concentration (*v*/*v*), and (**c**) pH were determined at different time points during bottle conditioning for the different yeast strains. Light purple: Ale brewing yeast 1; dark purple: Ale brewing yeast 2; green: Lager brewing yeast 1; orange: Lager brewing yeast 2; blue: Sparkling wine yeast; red: Distilling yeast. Data shown as the mean and error bars represent standard deviation of biological replicates.

To obtain a better understanding of the influence of the different yeast strains on the final beer product, characteristics such as AE, alcohol concentration, and pH were evaluated for up to 120 days. We found that while the AE decreased, the alcohol concentration increased until day 14 of bottle conditioning (Supplementary Figure S4a,b). This was expected as the yeast utilises the fermentable extract and produces ethanol through fermentative metabolism. After the yeast reached the stationary phase, the rate of AE consumption and alcohol production were minimal as the yeast cells entered a non-proliferative state and downregulated processes for active growth [21]. The pH of the beer decreased for all samples (Supplementary Figure S4c), which was expected because of the excretion of organic acids that typically occurs during fermentation [22]. Beyond 14 days, the AE of Lager brewing yeast 2 was lower than all other strains at all time points (Figure 2a). A similar trend was observed for the alcohol concentration, which was higher in beer conditioned with Lager brewing yeast 2 until day 60 (Figure 2b). This suggested that the Lager brewing yeast 2 had improved fermentation performance compared to the other strains. Compared to days 14 and 30, Ale brewing yeast 2 and Lager brewing yeast 2 showed a lower AE and higher alcohol concentration at day 60 of bottle conditioning. This suggests that these strains required more time to fully referment the beer. The ability of Ale brewing yeast 2 to referment the beer at a low cell concentration could indicate that successful bottle conditioning does not require high cell concentrations. Generally, the pH of beer increased from day 60 for all yeast strains during bottle conditioning (Figure 2c). Overall, all strains were able to perform secondary fermentation of the beer.

### 3.3. Yeast Viability and Its Impact on Foam Stability during Bottle Conditioning

**Figure 3:**
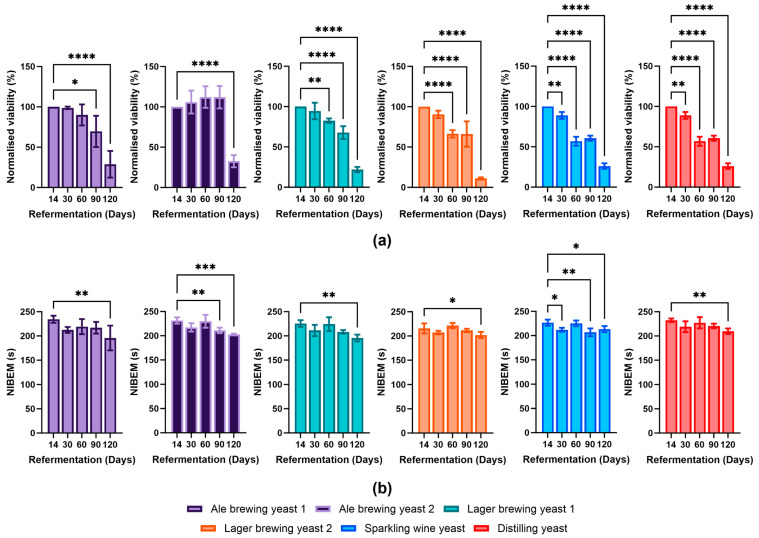
Impact of bottle conditioning on yeast survival and foam stability over 120 days. The (**a**) viability (normalised to day 14) and (**b**) foam stability of the different yeast strains was determined at day 14–day 120 of bottle conditioning. Data shown as the mean value and error bars represent standard deviation. Light purple: Ale brewing yeast 1; dark purple: Ale brewing yeast 2; green: Lager brewing yeast 1; orange: Lager brewing yeast 2; blue: Sparkling wine yeast; red: Distilling yeast. Statistical tests were performed for each strain using an ordinary, one-way ANOVA. * *p*-value ≤ 0.05, ** *p*-value ≤ 0.01, *** *p*-value ≤ 0.001, **** *p*-value < 0.0001.

The viability of the different yeast strains was determined over four months of bottle conditioning (Figure 3a). At day 30 of bottle conditioning, the Sparkling wine strain showed the largest decline in viability amongst all the strains (89 ± 4%) when compared to day 14. The Ale brewing yeast 1 (99 ± 2%), Ale brewing yeast 2 (106 ± 14%), Lager brewing yeast 1 (95 ± 10%), Lager brewing yeast 2 (91 ± 5%), and Distilling yeast (98 ± 1%) did not show a large decrease in viability. At day 60, all yeast strains (except for Ale brewing yeasts 1 and 2) began to decline in viability. The viability of Lager brewing yeast 2 and Sparkling wine yeast decreased considerably, obtaining viabilities of 66 ± 5% and 57 ± 6%, respectively. Interestingly, the viability of Ale brewing yeast 2 did not demonstrate any decline until the day 120 measurement. All yeast strains by day 120 possessed a low viability (below 30%), with a significant proportion of the yeast cells expected to have autolysed [23]. Overall, we observed that the Ale brewing yeast strains and Lager brewing yeast 1 were able to maintain their viability for longer compared to the Lager yeast strain 2, Sparkling wine, and Distilling yeast strains, with Ale brewing yeast 2 outperforming all yeast strains.

The foam stability of beer can be impacted by a decrease in yeast viability. For example, the enzyme Proteinase A can be secreted into the beer during yeast autolysis, resulting in cleavage of foam stabilising compounds and a decrease in foam stability [24,25]. The viability of the Sparkling wine yeast significantly decreased from day 30. This corresponded with a faster decline in viability. Interestingly, Ale brewing yeast 2 showed a decline in foam stability after day 60 despite no decline in viability. There were decreases in foam stability observed for all yeast strains at day 120 of bottle conditioning, some of which were statistically significant compared to day 14 (Figure 3b). As the viability of all yeast strains at day 120 was below 30%, it is likely that yeast autolysis contributed to the decreased foam stability [23]. It seems that for most of the strains utilised in this study, the foam stability was not impacted until there was a severe decline in viability. As a decrease in foam stability did not correspond with a decrease in viability for all yeast strains, it indicated that the secretion of proteolytic enzymes into the beer was strain-specific. Therefore, consideration of the yeast strain for bottle conditioning is important as some strains are more likely to impact foam stability than others.

### 3.4. Impact of Yeast Strain on Volatile Profile

**Figure 4:**
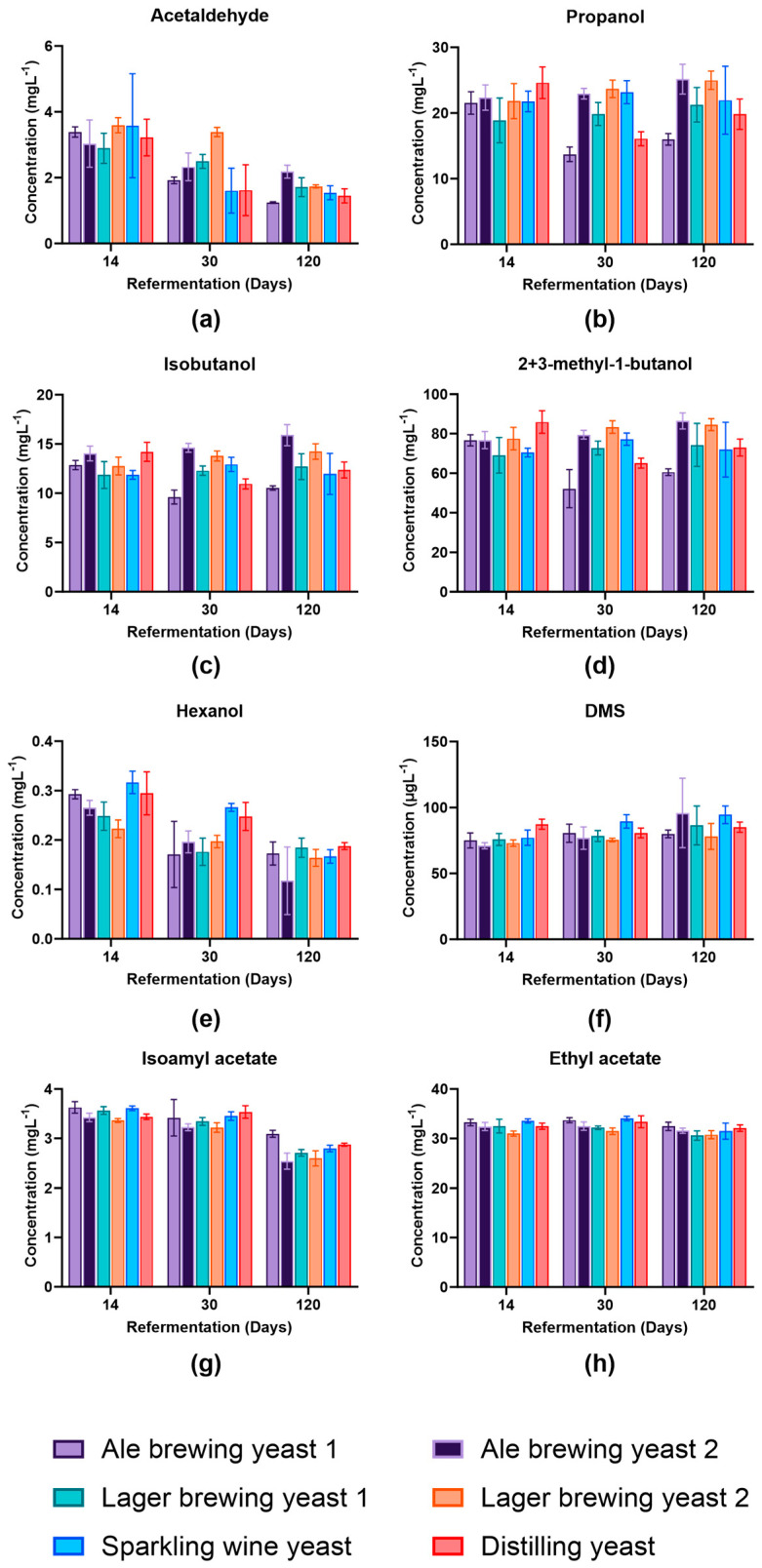
Concentration of volatile compounds produced by different yeast strains at various stages of secondary fermentation in beer. Concentration (ppm) of (**a**) acetaldehyde, (**b**) propanol, (**c**) isobutyl alcohol, (**d**) 2-methyl-1-butanol and 3-methyl-1-butanol, (**e**) hexanol, (**f**) dimethyl sulfide (DMS) in µgL^−1^ (ppb), (**g**) isoamyl acetate, and (**h**) ethyl acetate. Data shown as the mean value and error bars represent standard deviation. Light purple: Ale brewing yeast 1; dark purple: Ale brewing yeast 2; green: Lager brewing yeast 1; orange: Lager brewing yeast 2; blue: Sparkling wine yeast; red: Distilling yeast.

The aim of this evaluation was to produce a beer with a yeast that had enhanced viability but did not alter the profile of the beer to preserve product identity. The concentration of eight volatile compounds was assessed at day 14 to determine whether the different yeast species changed the volatile distribution compared to that expected for a pale ale. This was performed at day 14 to align with the standard quality control practices within the brewery. The concentration of acetaldehyde, propanol, isobutanol, and 2-methyl-1-butanol/3-methyl-1-butanol (2+3-MB) were similar at day 14 between yeast strains (Figure 4a–d). Generally, the concentration of dimethyl sulfide (DMS), ethyl acetate, and isoamyl acetate remained similar between yeast strains at the different time points analysed (Figure 4f,g).

The acetaldehyde concentration decreased during secondary fermentation. This was expected as acetaldehyde is a precursor for ethanol formation [26]. The concentration of acetaldehyde was highest in Lager brewing yeast 2 at day 30; however, on day 120, it was at a similar concentration to all the other strains except Ale brewing yeast 1. This reflected the ability of different strains to metabolise the acetaldehyde [27]. Propanol, isobutanol, and 2+3-MB were consistently lower in Ale brewing yeast 1 at day 30 and day 120 and Distilling yeast at day 30 compared to the other yeast strains. Higher alcohols such as propanol, isobutanol, and 2+3-MB are formed as a by-product of yeast amino acid metabolism from keto-acids via the Ehrlich pathway [26]. The lower concentration in Ale brewing yeast 1 may indicate that this strain was more efficient at utilising residual amino acids present in the beer [27]. Hexanol concentrations were higher in Sparkling wine yeast and Distilling yeast at day 14 and day 30 and was most likely strain-dependent [27]. However, the reduction in the content of hexanol during bottle conditioning most likely occurred due to the formation of the corresponding ester, hexyl acetate, as a product of yeast metabolism [28]. Isoamyl acetate concentration decreased in concentration by day 120 of bottle conditioning for all yeast strains. A decrease in isoamyl acetate also occurred during beer ageing and was associated with a decrease in fruity flavour and increasing the perception of eventual stale flavours [29].

Overall, the concentration of the main volatile compounds was relatively similar between yeast strains in this study. We performed a sensory evaluation to determine whether the different yeast species resulted in an aroma typical for a pale ale (Supplementary Table S2). The panel was asked to comment on the sensory aroma of the beer compared to a typical pale ale profile and to select whether the product was typical (or atypical) for a pale ale beer. The use of Sparkling wine yeast and Distilling yeast resulted in a different aroma profile to the standard profile expected, so they were categorised as atypical. The panel commented that this was mainly due to the presence of 4-vinyl guaiacol. Ale brewing yeast 1 (the yeast normally used for bottle conditioning at the brewery) was consistently selected as typical, with at least 60% of the panellists stating it possessed typical characteristics across all time points. Interestingly, Ale brewing yeast 2 was initially classified as atypical by the panellists but subsequently found to be unanimously typical from day 30 to 90. This indicated that Ale brewing yeast 2 required a longer bottle conditioning time to obtain similar characteristics as the control beer. In addition, 60% of the panellists found the product using Ale brewing yeast 2 was atypical at day 120. Lager brewing yeast 1 and Lager brewing yeast 2 had similar typical rankings throughout bottle conditioning. Interestingly, Ale brewing yeast 1, Lager brewing yeast 1, and Lager brewing yeast 2 were ranked as typical by 100% of the panellists at day 120 despite a decline in viability. These results show that a similar sensory profile was obtained when any of the brewing yeast strains were utilised for bottle conditioning.

### 3.5. Proteomic Profiling to Investigate Key Protein Changes That Are Associated with Decreased Viability

In order to determine the biological changes that allow specific yeast strains to maintain their viability, we performed proteomic analysis of Ale brewing yeast 2 (superior viability) compared to Sparkling wine yeast (poor viability) from day 14 to day 120 using Zeno Sequential Window Acquisition of All Theoretical Mass Spectra (Zeno SWATH-MS). Day 14 was chosen as the control in this study as the yeast strains were all in stationary phase at this time point and it was prior to the onset of a decline in viability. Our proteomic analysis resulted in the quantification of 2383 ± 219 proteins across all samples (Supplementary Table S3).

**Figure 5:**
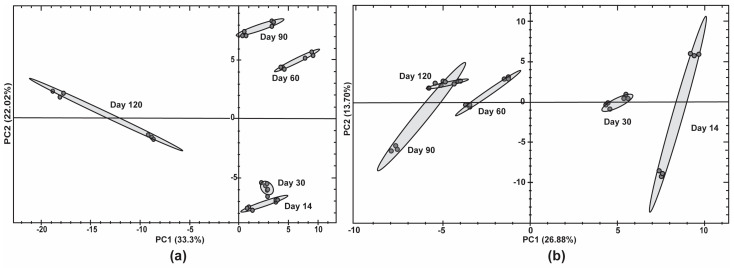
Proteomic profiling of proteins during late stage of bottle conditioning in beer using different yeast strains. Principal component analysis (PCA) of the normalised protein abundances in (**a**) Ale brewing yeast 2 and (**b**) Sparkling wine yeast using Spectronaut 18 software.

Principal component analysis (PCA) was performed to evaluate the major principal components of variance between the different days of late-stage secondary fermentation for Ale brewing yeast 2 (Figure 5a) and Sparkling wine yeast (Figure 5b).

Generally, the PCA grouped biological replicates by time point in the first principle component (PC1). PC1 showed a clear separation (33.3%) between early time points (day 14 to day 90) and the late time point (day 120) for Ale brewing yeast 2. The second principle component (PC2) separated (22.0%) the early time points (day 14 to day 90). In contrast, the PC1 for Sparkling wine yeast showed a clear separation (26.9%) between day 14 and day 30 compared to day 60 and day 90. PC2 had a small separation (13.7%) between biological replicates. The separation of PC1 clearly correlates to the ability of these strains to maintain their viability as Ale brewing yeast 2 and Sparkling wine yeast had a rapid decline in viability at day 120 and day 60, respectively. Therefore, the proteomic changes associated with the separation of secondary fermentation time may explain the different yeast strain’s ability to maintain viability during bottle conditioning.

To investigate this further, we evaluated significant changes in protein abundance (adj. *p*-value ≤ 0.05, Log_2_ fold-change ≥ 0.58) at each time point (compared to day 14) for Ale brewing yeast 2 (Supplementary Table S4) and Sparkling wine yeast (Supplementary Table S5). There were fewer significant protein changes at the earlier stage of bottle conditioning (day 30) compared to the later stages of bottle conditioning (day 60–day 120) in both yeast strains (Table 2).

Gene ontology analysis was performed to highlight the main biological functions that were altered in response for proteins that significantly changed in abundance. Interestingly, proteins involved in oxidoreductase activity and ribosome biogenesis were upregulated in Ale brewing yeast 2, whereas proteins associated with the cell wall were upregulated in Sparkling wine yeast (Table 3).

**Figure 6:**
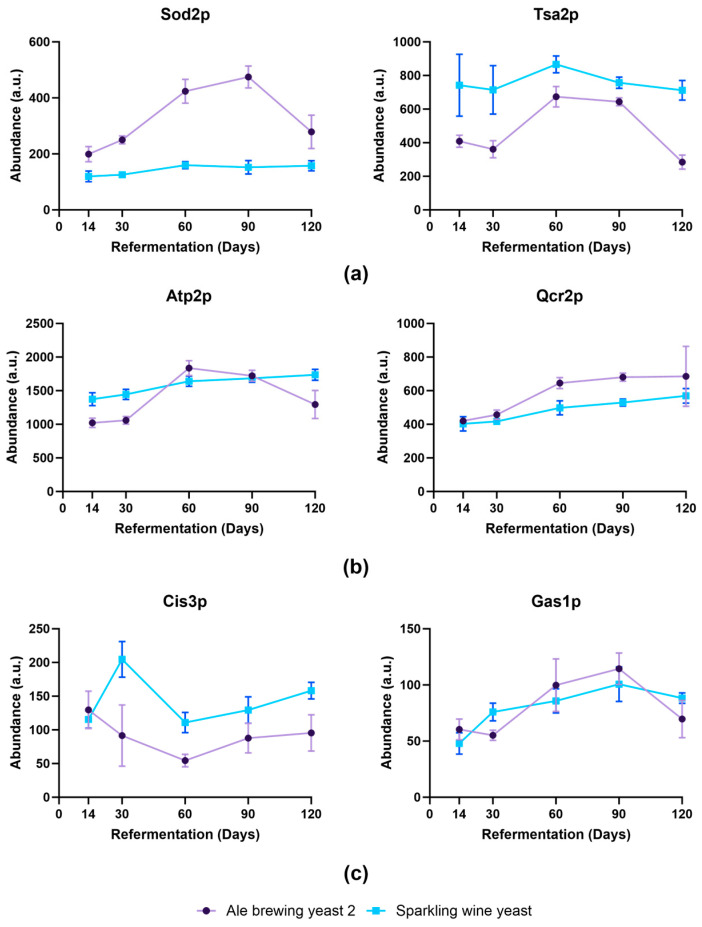
Specific examples of proteins that changed in abundance during bottle conditioning. Proteins shown were significantly enriched in GO term analysis (adj. *p*-value < 0.01) for different biological processes: (**a**) oxioreductase activity, (**b**) electron transport, and (**c**) cell wall organisation. Data shown as average abundance ± standard deviation of biological replicates. Protein abundance exported from Spectronaut export file. Purple: Ale brewing yeast 2; blue: Sparkling wine yeast.

Proteins associated with eliminating oxidative stress such as Sod2p, Ctt1p, Prx1p, Tsa1p, and Tsa2p increased in abundance in Ale brewing yeast 2 compared to Sparkling wine yeast, where their abundance remained similar (Figure 6a). An increase in expression of *PRX1*, *CTT1*, *SOD2*, and *TSA1* was shown to occur in yeast strains that had superior fermentation performance during wine-making [30]. This suggested that an increase in expression of oxidative stress genes enabled Ale brewing yeast 2 to respond better to stresses such as ethanol [30] or nutrient deprivation [31]. Additionally, proteins involved in the electron transport chain such as Qcr2p and Atp2p were also increased from day 60 in Ale brewing yeast 2 (Figure 6b). Mitochondrial genes, including *QCR2* and *ATP2*, were also found to increase in yeast during refermentation of wine [9,32]. There has been growing evidence that an increase in genes involved in mitochondrial function and oxidative metabolism is required in ethanol stress tolerance [33]. Therefore, the increase of proteins associated with these pathways suggested that Ale brewing yeast 2 was more tolerant to long-term ethanol exposure.

Proteins associated with the cell wall were increased in Sparkling wine yeast (Figure 6c). Cis3p, Pir1p, and Hsp150p only increased at day 30 in Sparkling wine yeast. In contrast, Cis3p and Hsp150p significantly decreased at day 120 and day 30, respectively, and Pir1p was not detected after day 14 in Ale brewing yeast 2. The increase in these proteins at day 30 in Sparkling wine suggested remodeling of the cell wall in response to stress. Other proteins associated with cell wall remodeling were altered during refermentation. For example, the abundance of Crh1p increased from day 30 in Sparkling wine yeast but was not detected in Ale Brewing yeast 2, whereas Crh2p was found to significantly increase in abundance at day 90 in Ale brewing yeast 2 compared to day 60 in Sparkling wine yeast. Crh1p and Crh2p are responsible for cross-linking β-1,3-glucan to chitin in the cell wall [34]. The expression of *CRH1* has been shown to increase in response to ethanol-induced cell wall stress [35,36]. Furthermore, the expression of *CRH2* is induced as a late response to perturbations of the β-glucan network [37]. The higher abundance of Crh1p and the earlier increase in abundance of Crh2p indicated that the Sparkling wine yeast was more susceptible to cell wall stress. Furthermore, the abundance of Gas1p was significantly increased at day 60 and day 90 for Ale brewing yeast 2 but at day 30 in Sparkling wine yeast. Gas5p abundance did not change in Ale brewing yeast 2 but decreased at day 120 in Sparkling wine yeast. Gas1p encodes a β-1,3-glucanosyltransferase that is responsible for modulating the branching and elongation of β-1,3-glucan chains [38]. As *GAS1* plays an important role in increasing cell wall stiffness in response to different stresses such as acidity, this further supports that Sparkling wine yeast was more susceptible to cellular stress [39]. The increase in abundance of these proteins may promote the earlier release of cell wall components and shorten the lifespan of this strain as genes involved in cell wall are upregulated in response to autolysis in brewing yeasts [40].

Proteins associated with the structural ribosome were downregulated during refermentation in both Ale brewing yeast 2 and Sparkling wine yeast (Table 4). Many of the proteins associated with the ribosome such as Rpl34Bp were only significantly decreased at day 120 in Ale brewing yeast 2 and Sparkling wine yeast. A decrease in proteins associated with protein synthesis typically occurs at the onset of stationary phase during primary fermentation and bottle conditioning [9,41]. As many of the ribosomal proteins would have decreased by day 14, the sudden decrease at day 120 was most likely associated with autolysis and the breakdown of proteins within the cell.

## 4. Conclusions

We demonstrated that six different types of yeast successfully performed secondary fermentation during the bottle conditioning of beer. Ale and Lager brewing strains were better for obtaining a standard beer aroma, while Sparkling wine and Distilling strains were more amenable to unique sensory flavour. Protein abundance changes from proteomics experiments revealed biological functions that contributed to the prolonged viability of Ale brewing yeast 2. Specifically, proteins associated with oxidoreductase activity and the electron transport chain enabled Ale brewing yeast 2 to respond to long-term ethanol stress. We anticipate that brewers could utilise a different yeast for bottle conditioning to improve beer longevity without detrimental effects on foam stability and the accompanying sensory profile. Future experiments will explore the effect of the pre-adaptation of yeast in beer or to specific environmental conditions such as high alcohol to extend their viability during bottle conditioning.

## Figures and Tables

**Table 1:** Commercial Saccharomyces strains used in this study.

Name	Strain	Taxonomic Name	Commercial Use	Supplied Format	Source
Ale brewing yeast 1	NA	*S. cerevisiae*	Primary ale fermentation and secondary fermentation of beer	Provided by agar slant	Coopers Brewery Ltd. (Regency Park, Australia)
Ale brewing yeast 2	NA	*S. cerevisiae*	Secondary fermentation of beer	Isolated by filtration from a commercial beer	Dan Murphy’s (Adelaide, Australia)
Lager brewing yeast 1	Nebulosa-TUM 66/70	*S. pastorianus*	Primary and secondary fermentation of beer	Provided by agar slant	Weihenstephan for Brewing and Food Quality, (Munich, Germany)
Lager brewing yeast 2	SafLager 34/70	*S. pastorianus*	Primary lager fermentation	Dried yeast	Coopers Brewery Ltd., (Regency Park, Australia)
Sparkling wine yeast	Lalvin EC-1118™	*Saccharomyces cerevisiae bayanus*	Secondary fermentation of sparkling wine	Dried yeast	Wine Quip, (Reservoir, Australia)
Distilling yeast	DistilaMax^®^ HT	*S. cerevisiae*	Neutral spirit fermentation	Dried yeast	Wine Quip, (Reservoir, Australia)

**Table 2:** Number of proteins with a significant abundance change during bottle conditioning *.

Condition	Upregulated		Downregulated	
	Ale Brewing Yeast 2	Sparkling Wine Yeast	Ale Brewing Yeast 2	Sparkling Wine Yeast
D30/D14	34	85	143	113
D60/D14	230	179	316	183
D90/D14	370	230	290	213
D120/D14	660	223	234	298

* Adj. *p*-value ≤ 0.05 and Log_2_ fold-change ≥ 0.58.

**Table 3:** Gene ontology of proteins that were significantly increased in abundance in Ale brewing yeast 2 and Sparkling wine yeast during bottle conditioning.

Ale Brewing Yeast 2				Sparkling Wine Yeast			
Term	*p*-Value *	Number of Proteins	Fold Enriched (%)	Term	*p*-Value *	Number of Proteins	Fold Enriched (%)
D30/D14							
No significant terms				Structural constituent of cell wall	2.98	4	4.7
D60/D14							
Catalytic activity	7.02	110	47.8	Glucosidase activity	2.93	7	3.9
Oxidoreductase activity	6.22	31	13.5	Hydrolase activity, hydrolyzing O-glycosyl compounds	2.74	9	5.0
Melatonin binding	4.81	6	2.6				
Electron transfer activity	3.33	8	3.5				
Proton transmembrane transporter activity	2.63	11	4.8				
Translation factor activity, RNA binding	2.13	8	3.5				
D90/D14							
Catalytic activity	9.11	168	45.4	Catalytic activity	3.43	99	43.0
Oxidoreductase activity	7.12	43	11.6	Primary active transmembrane transporter activity	2.45	11	4.8
Structural constituent of ribosome	3.72	31	8.4				
Electron transfer activity	2.59	9	2.4				
D120/D14							
Catalytic activity	5.87	104	46.6	Catalytic activity	5.87	104	46.6

* Adj. *p*-value (log_10_).

**Table 4:** Gene ontology of proteins that were significantly decreased in abundance in Ale brewing yeast 2 and Sparkling wine yeast during bottle conditioning.

Ale Brewing Yeast 2				Sparkling Wine Yeast			
Term	*p*-Value *	Number of Proteins	Fold Enriched (%)	Term	*p*-Value *	Number of Proteins	Fold Enriched (%)
D30/D14							
No significant terms				No significant terms			
D60/D14							
RNA polymerase III activity	4.29	8	2.5	No significant terms			
D90/D14							
5′-3′ RNA polymerase activity	2.91	9	3.1	No significant terms			
D120/D14							
Structural molecule activity	5.63	32	13.7	Structural molecule activity	9.61	44	14.8
Endopeptidase inhibitor activity	2.15	3	1.3				

* Adj. *p*-value (−log_10_).

## Data Availability

The mass spectrometry proteomics data have been deposited to the ProteomeXchange Consortium [16] via the PRIDE [17] partner repository with the dataset identifier PXD053930.

## Supplementary Materials

The following supporting information can be downloaded at: https://www.mdpi.com/article/10.3390/foods13162593/s1, Figure S1: Resazurin production during yeast propagation in wort; Figure S2: Viability of yeast strains at time of seeding for bottle conditioning; Figure S3: Cell concentration of different yeast strains during bottle conditioning; Figure S4: Beer characteristics during early stages of bottle conditioning; Figure S5: Concentration of volatile compounds produced by different yeast strains at various stages of bottle conditioning of beer; Table S1: Apparent extract and alcohol concentration after 72 h of propagation in wort; Table S2: Panellist classification of aroma profile of pale ale bottle conditioned using different yeast strains (%); Table S3: Average protein identifications across later stages of bottle conditioning in Ale brewing yeast 1 and Sparkling wine yeast; Table S4: Significant (adj. *p*-value) abundance changes (Log_2_ ≥ 0.58) in Ale brewing yeast 2; Table S5: Significant (adj. *p*-value) abundance changes (Log_2_ ≥ 0.58) in Sparkling wine yeast.
