# Supplementary material for: Performance of Different Saccharomyces Strains on Secondary Fermentation during the Production of Beer
Source: Foods. 2024 Aug 19;13(16):2593. doi: 10.3390/foods13162593 (PMC11354207; doi:10.3390/foods13162593)
Supplement: Supplementary file 1 [file foods-13-02593-s001.zip › Supplementary material.pdf]

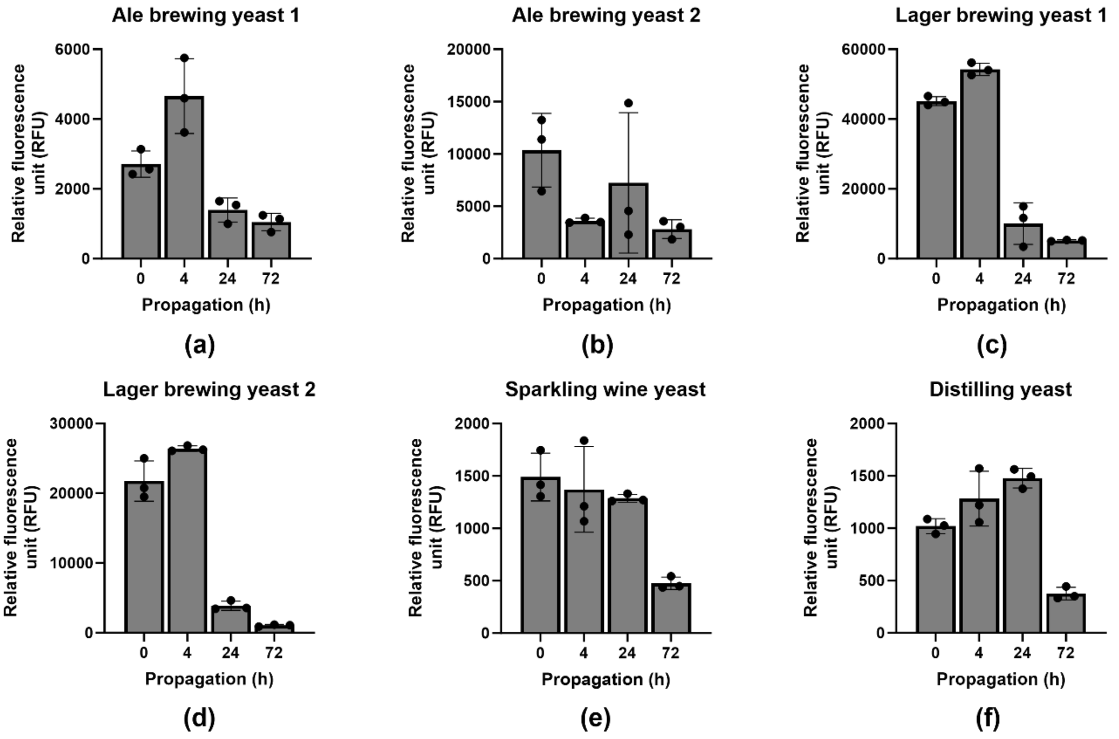

Supplementary Figure S1. Resazurin production during yeast propagation in wort. Yeast strains were cultured for 72 h in wort. 1x10^{6} cells were collected after 0, 4, 24, and 72 h and resuspended in 40 μM resazurin containing PBS. (a) Ale brewing yeast 1, (b) Ale brewing yeast 2, (c) Lager brewing yeast 1, (d) Lager brewing yeast 2, (e) Sparkling wine yeast, and (f) Distilling yeast. Data shown as the mean fluorescence of triplicate wells and error bars represent standard deviation.

Supplementary Table S1. Apparent extract and alcohol concentration after 72 h of propagation in wort

Yeast strain	Apparent extract (°P)	Alcohol concentration (*v*/*v*, %)
Ale brewing yeast 1	3.33 ± 0.12	4.69 ± 0.10
Ale brewing yeast 2	3.41 ± 2.33	4.08 ± 0.23
Lager brewing yeast 1	3.73 ± 0.32	4.62 ± 0.18
Lager brewing yeast 2	2.94 ± 0.02	5.06 ± 0.08
Sparkling wine yeast	5.04 ± 0.06	3.78 ± 0.05
Distilling yeast	4.49 ± 0.06	4.01 ± 0.02

Value expressed as mean ± standard deviation of duplicate measurements

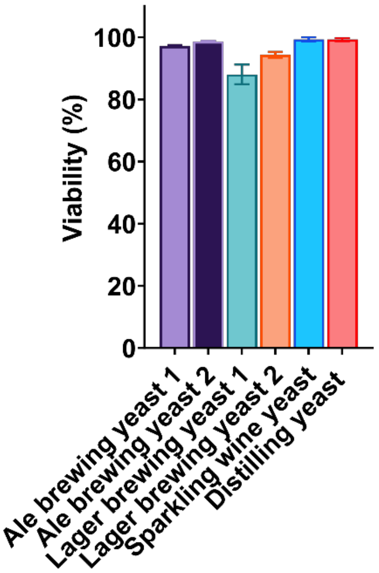

Supplementary Figure S2. Viability of yeast strains at time of seeding for bottle conditioning. Viability of yeast strains was determined using acridine orange and propidium iodide. Data shown as mean cell concentration and error bars represent standard deviation of duplicate measurements. Light purple: Ale brewing yeast 1; Dark purple: Ale brewing yeast 2; Green: Lager brewing yeast 1; Orange: Lager brewing yeast 2; Blue: Sparkling wine yeast; Red: Distilling yeast.

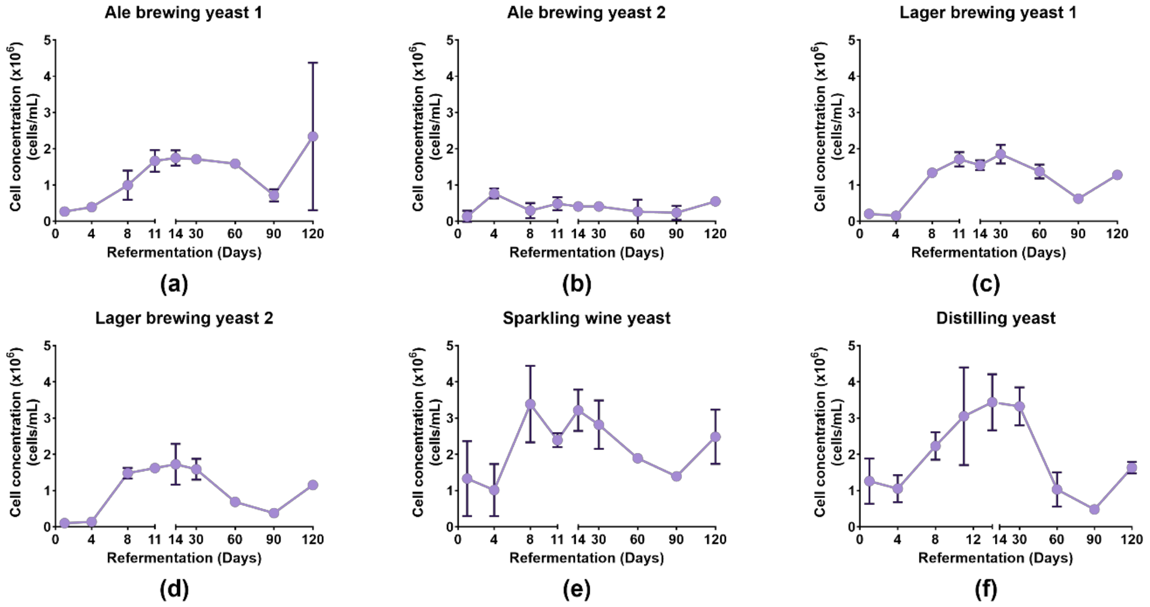

Supplementary Figure S3. Cell concentration of different yeast strains during bottle conditioning. Yeast strains were seeded into pale ale beer that was primed with 0.5 °P fermentable extract and incubated at 20 – 22 °C for 4 months. Cell counts were determined for (a) Ale brewing yeast 1, (b) Ale brewing yeast 2, (c) Lager brewing yeast 1, (d) Lager brewing yeast 2, (e) Sparkling wine yeast, and (f)

Distilling yeast. Data shown as the mean cell concentration and error bars represent standard deviation of biological replicates.

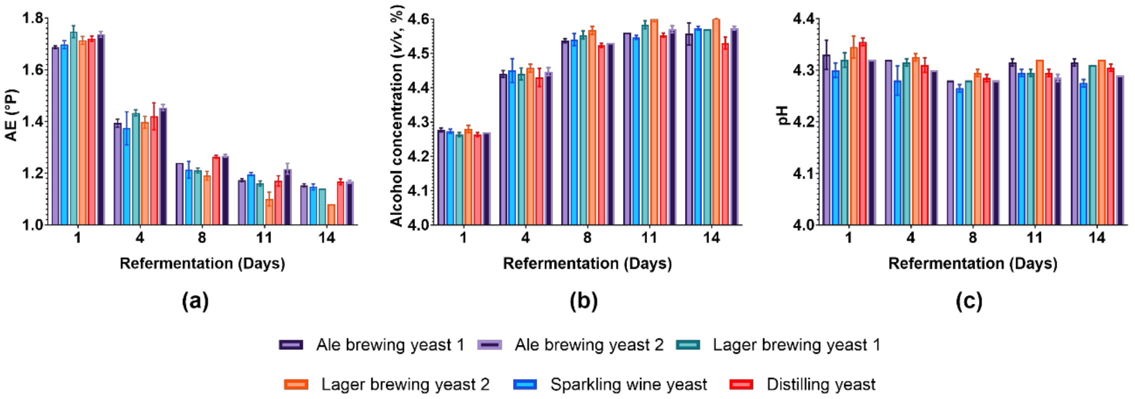

Supplementary Figure S4. Beer characteristics during early stages of bottle conditioning. (a) AE (°P), (b) alcohol concentration (*v/v*, %), and (c) pH were determined at different time points up to day 14 for different yeast strains seeded into pale ale beer. Data shown as the mean value and error bars represent standard deviation of biological replicates. Light purple: Ale brewing yeast 1; Dark purple: Ale brewing yeast 2; Green: Lager brewing yeast 1; Orange: Lager brewing yeast 2; Blue: Sparkling wine yeast; Red: Distilling yeast.

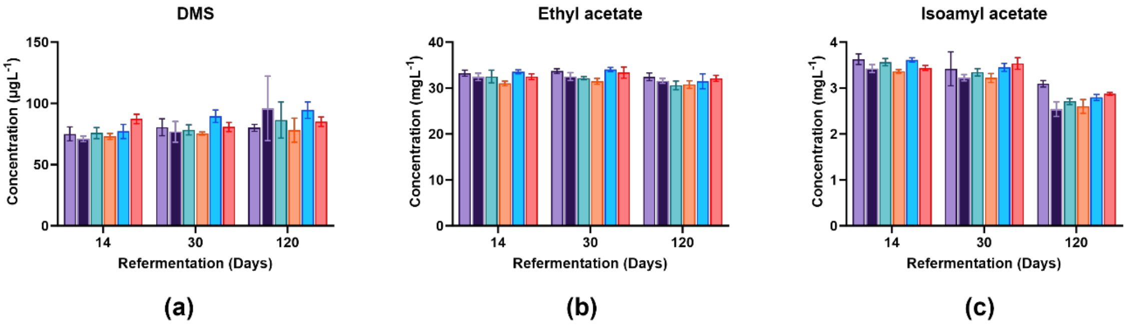

Supplementary Figure S5. Concentration of volatile compounds produced by different yeast strains at various stages of bottle conditioning of beer. Concentration of (a) dimethyl sulfide (DMS) in μgL^{-1}, (b) ethyl acetate in mgL^{-1}, and (c) isoamyl acetate in mgL^{-1}. Data shown as the mean value and error bars represent standard deviation. Light purple: Ale brewing yeast 1; Dark purple: Ale brewing yeast 2; Green: Lager brewing yeast 1; Orange: Lager brewing yeast 2; Blue: Sparkling wine yeast; Red: Distilling yeast.

Supplementary Table S2. Panellist classification of aroma profile of pale ale bottle conditioned using different yeast strains (%).

	Panellists describing aroma as typical for pale ale beer (%)[a]					
**Refermentation (Days)**	**Ale brewing yeast 1**	**Ale brewing yeast 2**	**Lager brewing yeast 1**	**Lager brewing yeast 2**	**Distilling yeast**	**Sparkling wine yeast**
14	75	0	62.5	87.5	0	0
30	60	100	100	60	0	0
90	75	100	62.5	62.5	0	0
120	100	40	100	100	0	0

	Panellists description of aroma					
**Refermentation (Days)**	**Ale brewing yeast 1**	**Ale brewing yeast 2**	**Lager brewing yeast 1**	**Lager brewing yeast 2**	**Distilling yeast**	**Sparkling wine yeast**
14	Pleasant esters aroma	Diacetyl, acetaldehyde aroma	Low ester profile	Clean and pleasant taste	Phenolic, 4-vinyl guaiacol	Phenolic, 4-vinyl guaiacol
30	Lower ester profile than expected	Nice ester profile	Low to moderate ester profile	Clean aroma and taste	Phenolic, wheat beer characteristics	Phenolic aroma
90	Ester aroma	Ester aroma slightly lower	Bready aroma	Bready aroma	Phenolic aroma	Phenolic aroma
120	Pleasant aroma	Harsh aftertaste	Pleasant aroma	Younger flavour profile	Phenolic, wheat beer characteristics	Phenolic aroma

[a]Number of panellists for each sensory evaluation: day 14: 8; day 30 30: 5; day 90: 8; day 120: 5.

Supplementary Table S3. Average protein identifications across later stages of bottle conditioning in Ale brewing yeast 1 and Sparkling wine yeast. Values represented as mean ± standard deviation of biological replicates.

Yeast strain	Refermentation (Days)	Protein identifications
Ale brewing yeast 1	14	2623 ± 12
	30	2672 ± 39
	60	2663 ± 37
	90	2296 ± 225
	120	2574 ± 243
Sparkling wine yeast	14	2170 ± 65
	30	2228 ± 20
	60	2222 ± 14
	90	2185 ± 19
	120	2199 ± 31
